# Mr. Hamilton's Case of Puerperal Fever

**Published:** 1810-08

**Authors:** W. Hamilton

**Affiliations:** Ipswich


### *To the Editors of the Medical and Physical Journal.*

GENTLEMEN,

HAVING observed many useful observations and histories of cases of midwifery in your valuable Journal, I beg leave to transmit you the history of a case, which although it terminated fatally, will tend to shew what has been done on the subject, and point out the necessity of endeavouring to find out some more effectual means in similar cases.

May the 28th. I was desired to visit Mrs. W——— with all possible speed, as she was then in labour. I arrived about eleven o'clock on the forenoon of Monday. I learned she had been in labour since two o'clock on Sunday morning, but it being her first child, and she not supposing herself farther advanced in pregnancy than five months, had not called any midwife sooner, thinking her pains would subside; some of her attendants even persuaded her that it was the colic, and she had obtained some spirituous medicine with a view to relieve herself; but finding this did not answer, she was induced to send for medical aid. I proceeded to examine her, and found the membranes ruptured (which had happened a few hours before) and the

 head head of a full grown child locked in the pelvis. The external parts were very little dilated and appeared very rigid, which I attributed to its being the first child and to her advanced age (for she was in her thirty-ninth year); the head had advanced in its natural position, and delivery seemed only prevented by the resistance of the external parts. As the pains were frequent and sharp I determined to remain with her a few hours, to ascertain what progress would be made; after which, finding no farther advance nor relaxation of the parts, I took about twelve ounces of blood from her arm and gave her forty drops of opium. This in some measure composed her between the pains, which however continued very violent. In the evening she complained of a frequent desire to make water, when the catheter was introduced and about a quart of water came away. With a view to farther relaxation, an emollient injection was thrown up, and she was ordered a pill of three grains of opium. I remained with her till twelve at night, as her throes continued much the same, and observing no farther advance, and that she was inclined to sleep between the pains, I took my leave for a few hours, desiring to be informed should any change take place.

On Tuesday morning, being still more harrassed with pain, I was called up. I again examined her, and finding that there was no farther advance, and that she was much exhausted by the continuance of the pains, I thought delivery must be attempted; and as the head was so low, the forceps appeared likely to be of service, could they be applied. At this time a continued pain of the abdomen and general soreness called for warm fomentations, which were persisted in for more than an hour, and at the same time a dose of opium was administered. This having somewhat alleviated the pain and restlessness, every thing was deferred for a few hours longer. Examining again, and observing no farther advance of the head, or dilatation, I endeavoured to introduce the extractor, which I accomplished with some difficulty, but was unable to make any progress even with it, as the rigidity of the external parts would scarcely admit of its employment. Fearing therefore lest I should irritate and inflame the parts, I made no farther attempt.

A consultation was now called, when embryotomy was proposed as the only means likely to save the mother, for there was little doubt but the child was dead, from the putrid discharge. As there was no flooding, and as the pains had somewhat subsided, it was judged advisable first to to give her another large dose (five grains) of opium, and apply fomentations to the external parts, with a view to relax them. This in some degree had the desired effect, but instead of producing rest, the pains appeared to come on with increased vigour, and she continued in this state till towards evening, when they again began to subside. At this time I examined, to ascertain whether those violent pains which had just subsided, had produced any advancement, when I found the head had become somewhat lower, and the parts were more dilated. The only pain which she now felt was a general soreness of the abdomen and a desire to void her urine.

The patient being placed in that position in which we usually draw off the water, I endeavoured to introduce the catheter, but found I could not, on account of the advance of the head. Thinking I could now apply my hands with more effect in this position, I insinuated the fore finger of my right hand along the occiput, and the same of the left over the os frontis, and continuing an undulatory motion for a few minutes, I effected, unexpectedly, the delivery of the head, which was much facilitated by its putrid state, causing it to give way, and admitting the bones to overlap each other. The delivery of the head being thus effected, the body immediately followed in a most putrid state.

Having happily succeeded thus far, our attention was next called to the delivery of the placenta. The hæmorrhage was not very great, but her strength being greatly exhausted, and as there were no pains, it was thought advisable to delay any attempt till she recovered. The umbilical cord was in such a putrid dissolved state that it came away, separating itself from the placenta, on using the gentlest force. She now became quite easy and had several sleeps during the night.

Wednesday morning she appeared tolerably well, and had not experienced any pains since last evening. The fœtor of the discharge was so very great that a fumigating lamp was obliged to be used, notwithstanding which the nurse was seized with a diarrhœa. It was now judged necessary that the placenta should be delivered, lest she should sink under a putrid fever. I therefore lubricated my hand, and began to insinuate my fingers in a conical form, within the os externum, which I found at first slipped into a laceration of the right labium. This being avoided I renewed the attempt, and desiring her to direct her inspiration, and bearing down at intervals, in imitation of pains, I accomplished its introduction. The os uteræ still remained sufficiently dilated to admit my hand, on passing which, the first thing I perceived was a quantity of coagulated blood, the greater part of which I brought away before I proceeded any farther. I then felt for the cake, which I discovered firmly adhering to the fundus uteri. By inserting my fingers between it and the uterus, and using gentle force, I soon completely separated it; and now grasping it in my hand, I cautiously withdrew it. The hæmorrhage at first was considerable, but soon became moderate. After a few minutes I again introduced my hand, to find whether any pieces had been left detached; but not finding any, I withdrew it, bringing away a considerable quantity of coagulated blood. I judged this precaution the more necessary as the placenta was in so putrid a state that it separated even by its own weight. Thus she was freed of the secundines just twelve hours after the birth of the child, since which the lochial discharge has been but small, and she appears quite easy and composed. She continued in a very favourable state since the delivery of the placenta on Wednesday morning until Friday afternoon, when she was attacked with a violent rigor, succeeded by pain and tension of the lower part of the abdomen. She was now ordered a dose of castor oil, and fomentations to the hypogastric region, with an anodyne in the evening.

Saturday morning much better; has had several stools; pain of the abdomen considerably abated. She has slept pretty well during the night; has had several slight rigors; appetite good, pulse rapid and full, with foul tongue and pain of the head; she was ordered a saline mixture and to abstain from all stimuli. In the evening every symptom recurred with redoubled violence, pulse rapid and strong, breathing difficult, pain excruciating, great restlessness, a continued diarrhœa, and complete obstruction of the lochia. Being thus seized with extensive inflammation, she was ordered to lose six ounces of blood, which although a small quantity, had nearly induced syncope. When she recovered she took a draught of opium and antimonial wine, which gave her some rest, and produced a gentle diaphoresis during the night; when she awoke in the morning she again seemed much revived, drank a cup or two of tea, and only complained of a general soreness and stiffness all over the abdomen, extending down the thighs.

On Sunday evening she became much worse: no appetite, debility so great that she was unable even to turn in bed; countenance became pale; the diarrhœa which had continued ed for two days now ceased. She was ordered to continue her saline mixture, and another sudorific draught was administered. The pulse had now sunk considerably, and putridity had much increased, so that not only the people about her, but even she herself, could scarcely support the fœtor, although the fumigating lamp was still kept at the bed side. I may just observe, that the weather was extremely warm during the whole time of her indisposition. At this time she was much harrassed with frequent retchings and vomitings. A large blister was now applied over the hypogastric region, and the antiseptic plan was now adopted instead of the antiphlogistic. With this view she took a desert spoonful of yeast every two hours, with an anodyne draught. Monday morning she appeared again much better, her retchings and vomiting having entirely subsided. She drank a cup of tea this morning, her appetite being much better than yesterday morning, which I attributed to the salutary effects of the yeast. The blister had not drawn, and it was therefore renewed. She gradually became worse during the day, so that nothing could now be taken but some porter, which was used as her common drink. All her pains having ceased, she appeared quite easy and slept a great deal. I now left her, not thinking she would survive till morning. Calling on Tuesday morning, I found her in a similar state. She was quite sensible at intervals, when the stupor subsided. She continued in a comatose state during the day, and survived till Wednesday morning, retaining her senses to the last, when she calmly passed into the extended arms of death.

Thus terminated this melancholy case on the eleventh day from its commencement. The membranes broke thirty two hours after labour began; the child was born sixty-eighty hours after, and thirty-six from the rupture of the membranes; placenta delivered twelve hours after the birth of the child, and about fifty-two before the fever commenced.

There is no doubt but the latter part of this case was the disease termed puerperal fever, which became epidemic in London in 1770, and which, I believe, was first particularly treated of as an original disease by Dr. Hulme. He describes it as a disorder which most commonly attacks the patient on the second or third day after delivery, when the belly becomes painful, tense, and sore to the touch, succeeded by rigors, pain in the head, heat, thirst, inquietude, and a quick pulse with difficult respiration and often retching and vomiting. Having inspected several

 bodies bodies who died of this disease, he found the omentum and intestines in a highly inflamed state. He therefore very properly attributes the cause to inflammation. In his indications of cure he places much more reliance on cathartics and diaphoretics than venesection, which he cautiously recommends.

I am, &c.

W. HAMILTON.

*Ipswich, June* 10, 1810.

